# The Burden of Hormonal Disorders: A Worldwide Overview With a Particular Look in Italy

**DOI:** 10.3389/fendo.2021.694325

**Published:** 2021-06-16

**Authors:** Andrea Crafa, Aldo E. Calogero, Rossella Cannarella, Laura M. Mongioi’, Rosita A. Condorelli, Emanuela A. Greco, Antonio Aversa, Sandro La Vignera

**Affiliations:** ^1^ Department of Clinical and Experimental Medicine, University of Catania, Catania, Italy; ^2^ Department of Health Sciences, University Magna Græcia of Catanzaro, Catanzaro, Italy; ^3^ Department of Experimental and Clinical Medicine, University Magna Graecia of Catanzaro, Catanzaro, Italy

**Keywords:** prevalence of endocrinopathies, incidence of endocrinopathies, epidemiology of the endocrine diseases, economic burden of endocrine diseases, clinical burden of endocrine disease

## Abstract

Endocrine diseases have a considerable impact on public health from an epidemiological point of view and because they may cause long-term disability, alteration of the quality-of-life of the affected patients, and are the fifth leading cause of death. In this extensive review of the literature, we have evaluated the prevalence of the different disorders of endocrine interest in the world and Italy, highlighting their epidemiological, clinical, and economic impact.

## Introduction

Data from the European Observatory of Health Policies and Systems show that in Italy cardiovascular diseases (40% in women and 35% in men) followed by oncological diseases (24% in women and 33% in men) represent the most frequent causes of subsequent mortality events. Endocrine diseases (5% in women and 4% in men) rank fifth on this list, after respiratory (6% in women and 8% in men) and neurological (8% in women and 5% in men) diseases (1). This impact on the risk of mortality can be attributed to the important systemic implications that many endocrine diseases have (**Table 1**).

**Table 1:** Systemic manifestations of the main endocrine diseases.

Disease	Systemic manifestations
**Hypothyroidism**	Bradycardia, diastolic hypertension, anemia, weight gain, hypercholesterolemia (2)
**Hyperthyroidism**	Tachycardia, systolic hypertension, atrial fibrillation, osteoporosis (3)
**Hypoparathyroidism**	Cardiac arrhythmias (QT prolongation), tissue calcifications (basal ganglia, nephrocalcinosis) (4)
**Primary hyperparathyroidism**	Osteoporosis, nephrolithiasis, renal failure, pancreatitis (5)
**Addison’s disease**	Hypotension, hypoglycemia, and electrolyte imbalances (particularly in forms also associated with mineralocorticoid deficiency) (6)
**Cushing’s syndrome**	Hypertension, diabetes mellitus, obesity, osteoporosis (7)
**Hyperaldosteronism**	Hypertension, electrolyte imbalances with hypernatremia and hypokalemia that can lead to fatal arrhythmias (8)
**Pheochromocytoma**	Hypertension, orthostatic hypotension, fatal arrhythmias, impaired glucose metabolism (9)
**Acromegaly**	Hypertension, hyperglycemia, heart failure, increased cancer risk, dyslipidemia (10)
**GH deficiency**	Increased visceral adipose tissue, risk of fracture, and atherogenic lipid profile, and decreased lean mass, skeletal muscle strength, cardiac capacity, BMD, and increased (11)
**Male hypogonadism**	Osteoporosis, impaired glucose metabolism, obesity, anemia (12)
**Diabetes mellitus**	Micro- and macrovascular complications (retinopathy, nephropathy, neuropathy, increased risk of cardiovascular events) (13)

Based on these assumptions, we conducted an overview of the prevalence and incidence of the main disorders of endocrine interest, highlighting their real impact on daily clinical practice. The data of prevalence and incidence were extracted, when possible, from national and international registers, such as the Istituto Nazionale di Statistica (ISTAT), the Istituto Superiore di Sanità (ISS), and ARNO Observatory, World Health Organization (WHO). When the former was not available, guidelines of international societies or reviews summarizing the epidemiological evidence collected in different geographical areas of the world and Italy were used. Many of the studies included were conducted in the last twenty years (2000-2020), but those conducted in the last four years (2017-2020) were preferred in the selection of the epidemiological data. This research strategy allowed us to extract the most recent epidemiological data available in the literature, although the lack of solid prevalence studies on some of the diseases addressed in some continents such as Africa, Asia, and Oceania only allows us to estimate the worldwide prevalence, but not to have a precise estimation.

## Pituitary Diseases

The most common pituitary disease is pituitary adenomas. Early diagnosis and eventual treatment of these masses are clinically essential as they can secrete hormones and cause a mass effect (headache, visual alterations, hydrocephalus). The general prevalence is about 1 case/865-2688 people even though autopsy studies report the presence of small pituitary adenomas in about 10% of people (14). Approximately, 50% of these masses are microadenomas (size <10 mm), while the rest are macroadenomas (size >10 mm). From a hormonal point of view, 32-66% of them are prolactinomas, 8-16% secretes growth hormone, 2-6% adrenocorticotropin (ACTH), and about 1% TSH (14). Non-functioning adenomas represent 15-54% of cases with a prevalence of 7-41.3/100,000 and an annual incidence of 0.65-2.34/100,000 inhabitants (15).

The other pituitary disease of clinical interest is hypopituitarism. This disorder is characterized by a partial or complete deficiency of pituitary hormone secretion resulting in variously combined clinical pictures of adrenal insufficiency (AI), hypothyroidism, hypogonadism, growth hormone deficiency, and less frequently diabetes insipidus. Patients with hypopituitarism, especially those with multiple hormonal deficits, represent an important source of health care costs and indirect costs (due to disability and workdays lost). In addition, they require the intervention of specialized centers to guarantee the best quality of life (QoL). The prevalence of the disorder is about 45 cases/100,000 with an annual incidence of 4 cases/100,000 inhabitants (16).

Among the diseases due to a reduced pituitary hormone secretion, GH deficiency (GHD) represents the most important endocrine cause of short stature with an estimated prevalence of 1:3500-1:4000 children. In ˜50% of cases, the deficit is idiopathic, in the remainder it is associated with hypopituitarism, central nervous system tumors, cranial irradiation, or other physiological causes (17). This disorder has also a significant clinical impact on adults. In fact, GH plays an important role in metabolism and bone, cardiovascular, and psychological health. Indeed, its deficiency is associated with decreased lean body mass with consequently decreased muscle strength and exercise endurance, decreased bone mineral density, increased fat mass, alterations in lipid profile and premature atherosclerosis, cardiac dysfunction, decreased fibrinolysis, and lower quality of life (QoL). Therefore, GH therapy is beneficial for adult patients with GHD to improve body composition, exercise capacity, skeletal integrity, lipid profile, and QoL. However long-term data on the prevention of the risk of fractures, clinical heart disease, and mortality are still lacking (18). For these reasons, GH treatment in adults must be individualized and requires careful monitoring of complications. In fact, the replacement therapy is contraindicated in the cases of active malignant diseases and proliferative diabetic retinopathy. Moreover, in diabetic patients, GH treatment may require adjustment of antidiabetic drugs. Finally, thyroid and adrenal functions should also be monitored because GH treatment may lead to a decrease in free thyroxine and cortisol serum levels (19).

However, the impact of GHD in the adult population is poorly understood as many children with GH deficiency are often not re-examined when they reach adulthood. Furthermore, the deficiency is often not sought in adult patients with risk factors such as vascular or traumatic brain injury. The annual incidence is estimated at 12 cases/1,000,000 inhabitants in France and 14-19 cases/1,000,000 inhabitants in Denmark. The scarce epidemiological information available today makes it necessary to increase the effort for the diagnosis and management of these patients (11).

AI due to ACTH deficiency is another dysfunction present in approximately one-third of the patients with hypopituitarism. It seems to have a higher prevalence than the primary forms estimated in about 150-280/1,000,000 inhabitants even if this figure is only based on few prevalence studies. Adrenal insufficiency caused by chronic glucocorticoid administration, which results in adrenal cortex atrophy due to suppression of the hypothalamic-pituitary-adrenal axis, is the one with the highest prevalence. However, to date, no data are available on its prevalence (20).

Pituitary disorders generally represent a significant clinical burden for the patient and an economic burden for the health care system since they are chronic diseases. In fact, even after treatment, the disease sequelae require medical treatment and compromise the patient’s QoL. For example, acromegalic patients may develop osteoarthritis, obstructive sleep apnea syndrome, hypertension that may persist even after surgical treatment. Even in Cushing’s disease, fractures, hypertension, and cardiovascular events constitute elements of long-term disability. Moreover, the side effects resulting from the administration of drugs used for the management of these diseases should not be overlooked. Finally, surgery and radiation therapy used for the treatment of these diseases can lead to hypopituitarism with all the consequences associated with this clinical condition (21).

## Thyroid Diseases

Thyroid dysfunctions are common and easily treatable. However, if undiagnosed, they can significantly affect health due to the vital role that thyroid hormones play in regulating heart and gastrointestinal function, brain development and function, physical development, and cellular metabolism (22). The most common thyroid disease is the nodule. Indeed, with ultrasound, the presence of thyroid nodules is found in 19-68% of the general population with a higher prevalence in women and the elderly. However, only 5% of nodular disease in women and 1% in men are palpable and therefore diagnosable at the physical examination. Finally, only 7% of thyroid nodules are found to be malignant on histological examination with a prevalence of 90% of the differentiated forms (papillary or follicular carcinoma) (23). Medullary thyroid carcinoma is much rarer, affecting 0.4-1.4% of subjects with a thyroid nodule. In 25% of cases, it can be an isolated familiar variant or associated with other neoplasms such as pheochromocytoma or adenoma/hyperplasia of the parathyroid glands giving the so-called multiple endocrine neoplasms 2 (MEN 2A or MEN 2B). The clinical course of the various forms differs being very aggressive in MEN2B, paucisymptomatic in a majority of patients with an isolated familiar variant, and with intermediate aggressiveness in patients with MEN 2A (24). The overall incidence of thyroid cancer in recent years has increased, probably also due to a better diagnostic capacity deriving from the use of ultrasound scans. In the United States, the incidence is about 14.3/100,000 inhabitants/year (23), while in Italy varies from 7 to 37.5/100,000 inhabitants/year depending on the region (25). Despite the high prevalence of the nodular disease, given the small percentage of nodules that turn out to be malignant and the low-risk phenotype of well-differentiated thyroid tumors, routine ultrasound screening is not recommended. Indeed, an over-diagnosis involves emotional consequences in the patients as well as potential risks related to over-medicalization and overtreatment. Moreover, over-diagnosis strains the capacity of health systems increases costs and diverts resources away from patients with greater health care needs (26).

The second most prevalent thyroid disease is chronic autoimmune thyroiditis, which is the leading cause of hypothyroidism in iodine-sufficient countries. The incidence of this disease is extremely variable depending on the geographical area, affecting from 30-150/100,000 people/year with a frequency 4-10 times higher in women than in men. The prevalence of hypothyroidism in patients with autoimmune thyroiditis is about 3.5-5/1000 in women and 0.6-1/1000 in men and it increases with advancing age (27). In Italy, it affects 5-15% of the female and 1-5% of the male populations.

Concerning subacute thyroiditis, lymphocytic and granulomatous forms are distinguished (28). The lymphocytic variant occurs more frequently in the postpartum but may also occur occasionally. It accounts for 29 to 50% of all cases of thyroiditis. The prevalence of postpartum thyroiditis varies from 1.1% in Thailand to 16.7% in Great Britain (29). In Italy, it has a prevalence of 5-9% of women giving birth (28). Subacute granulomatous thyroiditis is the main cause of a painful thyroid gland, generally caused by viral agents responsible for infection of the upper respiratory tract, such as echovirus, coxsackievirus, Epstein-Barr virus, influenza viruses, and adenovirus (30). Few epidemiologic studies have evaluated the prevalence of this form of thyroiditis. Of these, the largest is a community study performed in Olmstead County between 1960 and 1997. This study reported an incidence of 4.9 cases per 100,000 people per year (31).

Finally, a mention should be made the acute suppurative thyroiditis, an extremely rare condition but with a mortality rate of 7.8%. It accounts for 0.1-0.7% of all thyroid diseases. A recent systematic review that collected all the data published from January 2000 to January 2020 identified 148 studies on this topic, with a total of 200 cases described. The vast majority of the cases are due to bacterial infections (Streptococcus spp. and Staphylococcus spp). In countries with a high incidence of tuberculosis, acute tubercular thyroiditis is extremely frequent. Finally, in immunocompromised patients, forms caused by fungi can also occur with a mortality rate that can reach up to 33%. Among the predisposing factors, the most important is immunosuppression, followed by the presence of the pyriform sinus fistula, diabetes mellitus, and disseminated infection. Complications and sequelae include airway obstruction, dysphagia, esophageal perforation or fistula, Horner’s syndrome, an extension of the abscess resulting in mediastinitis, pericarditis, thrombophlebitis, sepsis, thyroid dysfunction associated with thyroiditis (hyper- or hypothyroidism), and death (32).

Another very prevalent thyroid dysfunction, affecting about 1-2% of the general population in iodine-sufficient countries, is ypothyroidism. In particular, the prevalence ranges from 0.2-5.3% in Europe and 0.3-3.7% in USA according to the definition used and the population studied (22). The main cause of this condition worldwide is still iodine deficiency. In iodine-sufficient regions, primary hypothyroidism can be ascribed to congenital or acquired causes, such as autoimmune or iatrogenic (secondary to thyroidectomy or radioiodine therapy). Secondary forms of hypothyroidism are rare and they are to a primary or secondary deficit of thyrotropin (TSH) secretion. The incidence of hypothyroidism has increased since 1995 from 3.5 cases/1000/year in women and 0.6 cases/1000/year in men to about 7 cases/1000/year, probably because of the higher number of patients undergoing thyroidectomy (33).

Finally, we have to consider hyperthyroidism that affects about 0.2-1.3% of the general population in iodine-sufficient countries, with an annual incidence of about 51 cases/100,000 inhabitants. The main cause of hyperthyroidism is represented by the Graves-Basedow disease, followed by toxic multinodular goiter and Plummer adenoma. Less frequent are instead the forms resulting from thyroiditis, TSH-secreting pituitary adenoma, and drug-induced forms (22). In Italy, a recent study has estimated a prevalence of hyperthyroidism of 756/100,000 inhabitants with an incidence of 81 cases per year per 100,000 people (34). It must be considered that often the treatment of hyperthyroidism requires invasive interventions such as surgery and radioiodine therapy that can lead to hypothyroidism. In particular, the latter is associated with the development of subsequent hypothyroidism in 10-20% of cases (35).

The socio-economic impact of thyroid diseases must not be overlooked. For example, goiter represents the 32nd most common sequelae of disease in humans. Furthermore, thyroid cancer is responsible for approximately 836,000 disability-associated life years (DALYs), while iodine deficiency represents the 85th major contributor to DALYs globally, with an even greater impact in some regions such as South Asia and Central Sub-Saharan Africa (36).

## Parathyroid Diseases

Chronic hypoparathyroidism is a rare clinical condition characterized by a deficiency in parathyroid hormone secretion that requires treatment with calcium salts and Vitamin D for more than 6 months. In most cases, it results from and unintentional iatrogenic damage to the parathyroid glands that can occur during thyroid surgery. Hypoparathyroidism caused by autoimmune disease or as a result of gene mutations is rare. Epidemiological studies on chronic hypoparathyroidism are lacking. A prevalence of 25-37 cases/100,000 has been estimated in the United States, while the prevalence in Denmark is 25.4 cases/100,000. In Italy, a Tuscan study estimated a prevalence of 27 cases/100,000, in line with the rest of the world (37). Chronic hypoparathyroidism has a major impact on the QoL of the affected patients. In particular, there is an inverse relationship between patients’ QoL and the disease symptom severity. The QoL is also influenced by patients’ compliance with the treatment often influenced by the cost and the need to take several tablets per day. In this sense, the administration of recombinant human parathyroid hormone has been seen to be correlated to lowers serum phosphate levels and a lower need for supplementation with calcium and vitamin D to maintain normocalcemia. However, it is still unclear whether this therapy has a better effect on the QoL of these patients, so further studies are needed to better explore this issue (38).

On the other hand, the prevalence of primary hyperparathyroidism (PHPT) is significantly higher, with 3 cases/1000 in the general population, increasing to 21 cases/1000 in the population aged 55-75 years (39). PHPT is associated with impaired bone health and an increased cardiovascular risk. Furthermore, patients with PHPT are more prone to the development of nephrolithiasis because of hypercalciuria that may accompany the disorder. In particular, it has been found that fractures in patients with PHPT occur although their bone mineral density (BMD) is ​​higher than those of women with postmenopausal osteoporosis. This is probably due to the microarchitectural changes that hyperparathyroidism causes and which are not identified by bone mineral densitometry. This requires careful evaluation even of those forms of mild hyperparathyroidism that often undergo a simple clinical follow-up. About the QoL of these patients, the evidence suggests that even mild hyperparathyroidism is associated with a worse QoL than that of healthy controls. However, no studies have clearly shown that surgery improves this parameter. Therefore, to date, the only worsening of the QoL does not represent a sufficient criterion for surgical treatment (38).

## Adrenal Diseases

The most prevalent adrenal disease is adrenal incidentaloma. By definition, adrenal incidentaloma is an adrenal mass that is incidentally diagnosed during imaging performed for other reasons. Autopsy studies estimate the prevalence of this disease to be around 2% of the general population ranging from 1% to 8.7%. The frequency increases with advancing age. Instead, the prevalence is 3% with an increase of up to 10% in the older population in the context of imaging diagnosis (40). In 75% of cases, incidentalomas are non-functioning adenomas, in 12% they responsible for Cushing’s syndrome, in 7% they are pheochromocytomas, and 2.5% are aldosteronomas, 5% metastasis (40). Finally, 8% of the adrenal masses are represented by adrenal carcinomas; this latter has an annual incidence of 0.7-2 cases/1,000,000 people (41).

Congenital adrenal hyperplasia (CAH) is a group of disorders caused by the mutation of one of the genes that encode steroidogenesis enzymes. CAH recognizes the deficiency of the 21α-hydroxylase enzyme in 90% of cases. Depending on the type of mutation and the enzyme involved, the clinical presentation of the disease is extremely variable from severe and life-threatening forms with salt loss crises (8.8 cases/100 patients) to virilizing forms (2.5 cases/100 patients) (41, 42). The prevalence of classic forms of CAH is about 1 case/15,000 people (34) with an annual incidence of an adrenal crisis of about 5.8 cases/100 patients (33). In Italy, a prevalence of 1 case/13,000-22,000 is estimated (42). Additionally, there is also a non-classical form of CAH that is diagnosed in adulthood due to the low degree of loss-of-function caused by the genetic mutation. The prevalence of this form is higher than that of classic variants, reaching 1 case/1,000 in the Caucasian population (42).

Another important condition is Addison’s syndrome. This disorder affects approximately 82-144/1,000,000 inhabitants with an annual incidence of 4.5 cases/1,000,000 (43). In the past, this condition was closely related to tubercular infection, while today the main cause is autoimmune that can be sporadic or associated with other endocrine and non-endocrine autoimmune diseases forming the so-called autoimmune polyendocrine syndrome (APS). Conversely post-infectious or genetically determined forms of Addison’s syndrome are rarer (43). Adrenal crisis is the most severe clinical manifestation of Addison’s disease with an annual incidence of 6-8 crises/100 patients with Addison’s diseases and a death rate of 0.5% (41). In Italy, the prevalence of Addison’s syndrome is around 117 cases/1,000,000 inhabitants (43).

Given the chronic nature of AI, the economic and social costs are very high. In fact, it is associated with a decreased QoL, absenteeism resulting in reduced productivity, premature mortality, and long-term morbidity including cardiovascular disease, infections, anxiety, and depression. In addition, they require constant monitoring of the therapy to avoid over-treatment associated with comorbidities such as obesity, osteoporosis, and impaired glucose tolerance. Finally, the diagnostic delay of AI must also be taken into account as the diagnosis is made at the onset of an adrenal crisis (44). A UK study conducted on 10,000 patients with AI divided into three groups (primary and secondary AI, and CAH), showed that patients with AI are responsible for costs ranging from about $8,000 to $32,000 per year per patient, with a higher economic cost for secondary forms and in patients with poor adherence to therapy. Furthermore, patients with AI have more frequent hospitalizations, with 8 to 10 times longer hospital days than matched controls (44).

The opposite condition characterized by endogenous cortisol overproduction is Cushing’s syndrome. The prevalence of this disease is about 10-15 cases per 1,000,000 inhabitants (45) and the incidence is 0.7-2.4/1,000,000 inhabitants/year (46). Cushing’s syndrome can be classified into ACTH-dependent (75-80% of cases) and ACTH-independent forms (15-20% of cases). The former is, in turn, due to the presence of ACTH-secreting pituitary adenomas (Cushing’s disease) in 75-80% of cases; an ectopic production of ACTH (pulmonary microcarcinoma, neuroendocrine tumors, etc.) is present 15-20% of the cases, and CRH-dependent forms have a prevalence of <1%. On the other hand, ACTH-independent Cushing’s syndrome is caused in 90% of cases by adrenal tumors, 80% of which are adenomas and the remaining carcinomas. There are also other rare forms of adrenal Cushing’s syndrome, such as macronodular adrenal hyperplasia, primary pigmented nodular adrenal disease (sporadic or as part of the Carney complex), and McCune-Albright syndrome (46). Cushing’s syndrome is responsible for an important clinical burden due to numerous morbidities, increased mortality, and reduced QoL. Mortality in turn is significantly associated with age at diagnosis and duration of hypercortisolism since comorbidities, such as metabolic syndrome and hypertension, are responsible for an independent increased cardiovascular risk. This implies the importance of an early diagnosis to prevent the occurrence of complications and thus future morbidity and mortality. Regarding the perceived well-being, patients present a significant reduction in QoL as a consequence of the numerous comorbidities (skeletal effects, metabolic syndrome, hypertension, neuropsychological disorders, increased infectious risk). In fact, patients with a late diagnosis present a reduction in QoL even after resolution of symptoms after surgery, testifying to the need for early intervention and better therapeutic management of these patients (47). The economic impact of the disease is also considerable. In fact, despite its rarity, an American study has shown that the annual cost of management of patients with Cushing’s disease is about twice that of a patient with diabetes mellitus (DM), and about four times that of patients without Cushing’s disease (48).

## Gonadal Diseases, Sexual Dysfunction, and Infertility

### Male Gonadal Diseases and Sexual Dysfunction

Male hypogonadism is a disorder characterized by reduced testicular function resulting in a deficient secretion of androgens, inhibin B, anti-müllerian hormone (AMH), and spermatogenic failure. The clinical features vary according to the age of hypogonadism onset. Before puberty, an impaired testicular function can be diagnosed based on low AMH and inhibin B levels. At the time of puberty, hypogonadism manifests as a pubertal delay. Congenital hypogonadism may become clinically manifest at birth with genital abnormalities (1 case/4500 births). The main cause of genetically determined primary hypogonadism is represented by Klinefelter syndrome with a prevalence of 1/500-1000 births. Among the acquired forms there are those secondary to chemotherapy and radiotherapy and functional forms associated with chronic diseases that have an annual incidence of 1/10,000-100,000. The most prevalent acquired form, closely related to age, is represented by late-onset hypogonadism (LOH). This condition affects about 2.1% of the male population aged 40-79 years and is related both to the physiological decline of testosterone production that occurs throughout life, the weight gains, and the development of chronic diseases that often characterize the elderly (49). Another acquired form of hypogonadism is that secondary to orchiectomy for testicular tumor. This accounts for 1-2% of all malignancies in men and is the most common malignancy among young men. The incidence ranges from 0.5-9.2/100,000 people (50). In Italy, the incidence is 7 cases per 100,000 inhabitants per year (51). Given the close association with comorbidities such as obesity, DM, and increased cardiovascular risk, hypogonadism is associated with poor clinical outcomes and high healthcare costs. In fact, testosterone deficiency appears to be associated with a higher complication rate and mortality risk in patients with DM type 2 and even patients who have major cardiovascular events seem to have a worse clinical outcome if concomitantly they have hypogonadism. Healthcare costs are due both to hypogonadism itself and the worsening of the comorbidities associated. Finally, the patient’s QoL is compromised both from a physical [asthenia, osteoporosis, worse urinary symptoms, and erectile dysfunction (ED)] and from a psychological point of view (52). This evidence imposes the need for an adequate assessment of the presence of hypotestosteronemia particularly in men over the age of 40 with comorbidities associated with this condition, such as cardiovascular disease, DM, osteoporosis, obesity, and depression. Furthermore, its treatment improves the QoL, clinical outcomes and decreases health costs (53).

The most prevalent male sexual dysfunction is premature ejaculation (PE). This affects about 30% of the male population worldwide (54). In Italy, the prevalence is around 20% in men over 18 years of age, however, only around 9% of them seek medical advice (55). The identification and treatment of men with PE is necessary given the great psychological impact not only on the patients affected but also on the female partners. In fact, the women partners of patients with PE often report lower sexual satisfaction, and higher personal distress and interpersonal difficulty (56). The other sexual dysfunction with great psychophysical impact is represented by ED, with an incidence of 12.4/1,000 cases per year in men aged 40-49 years, 29.8/1,000 in men aged 50-59 years, and 46.4/1,000 in men aged 60-69 years (57). The prevalence is about 20% in men younger than 30 years, 25% in men of 30-39 years, 40% in 40-49 years, 60% in 50-59 years, 80% in 60-69 years, and 90% in men older than 70 years (58). In Italy, the average prevalence of the disorder is about 12.8% (59). About 70% of them do not receive any treatment (60). The importance of identifying ED arises not only from the great psychological impact that it has but also from the significant clinical implications associated with it. In fact, it is now well-known that ED represents a sign for the development of future major cardiovascular events or DM, diseases with great clinical-socio-economic impact. Therefore, the effort in attempting to early diagnose these patients and correct their risk factors is essential (56).

### Female Gonadal Diseases and Sexual Dysfunction

The onset of ovarian dysfunction under the age of 40 years is called premature ovarian failure (POF) and it has a prevalence of 1%. POF is mainly due to autoimmune causes or genetic abnormalities. There are also acquired forms resulting from chemotherapy, radiotherapy, ovariectomy for ovarian cancer (61). This latter has an annual incidence of 15.2 cases per 100,000 women in Italy (51).

The ovarian dysfunction of the fertile age with the highest prevalence is represented by polycystic ovary syndrome (PCOS). This disorder has a considerable clinical impact because it not only causes menstrual irregularities, hyperandrogenism, and polycystic ovaries but can also be associated with alterations of glucose (insulin-resistance and impaired glucose tolerance) and lipid metabolism, which in turn are important cardiovascular risk factors (62). Women with PCOS have a 4-fold increased prevalence of DM type 2 and a 2.8-fold increase in gestational DM. Moreover, 20% of PCOS women develop DM before the age of 40 years (63). PCOS affects about 7% of women of childbearing age (64), although it is estimated that 75% of these women are not diagnosed during physician counseling, probably because of the extreme variability of PCOS clinical manifestations (62). In obese women, the prevalence of PCOS is even higher reaching 15-30% (62).

Sexual dysfunctions have a great epidemiological impact. It is estimated that about 40% of women worldwide have sexual dysfunction, but only 12-25% of them cause personal distress (65). The most prevalent dysfunction is represented by hypoactive sexual desire, which in the United States affects 39% of women (66) while in Europe it is reported to be prevalent in 29% of women (67). The other disorders with higher prevalence are low arousal (26% of women) and orgasm problems (21%) (66). These disorders are partly present also in the peri- and post-menopausal period. Each year, the number of menopausal women increases by approximately 47,000, and it is estimated that by 2030 there will be approximately 1.2 billion menopausal women (68). Eighty percent of these women present with complaints related to the hormonal changes associated with this condition (genitourinary symptoms, vasomotor symptoms, cognitive symptoms), but medical assistance is sought in only 25% of the cases (69).

The economic burden of female sexual dysfunction is poorly studied. However, significant is the impact in terms of anxiety, depression, interpersonal difficulties, broken relationships, and difficulties to conceive. All these aspects in turn contribute to increasing the economic impact of sexual dysfunction. Therefore, increased attention should be reserved for the diagnosis and treatment of these dysfunctions (56).

### Couple Infertility

Another condition of great endocrine interest and partly related to the previously listed disorders is represented by infertility. It is estimated that 15% of couples are infertile (about 48.5 million couples worldwide). Infertility recognizes a male factor in about 30% of cases, a female factor in 50%, and both partners are involved in the remaining 20% of cases (55). Therefore, in total, the percentage of men affected by infertility varies from 2.5 to 12% with more than 30,000,000 infertile men worldwide (70). On the other hand, it is estimated that about 2% of women aged 20-44 years have primary infertility, and another 10.5% of women of childbearing age experience secondary infertility (71). While a lot is known about female infertility, male infertility remains a fairly unknown area. In fact, although many causes of male infertility have been identified, up to now 50% of infertile patients do not receive an etiological diagnosis; thus these forms are defined as idiopathic infertility. In these cases, empirical treatment and assisted reproductive techniques (ART) are often the only therapy that can be proposed to overcome infertility regardless of the underlying mechanisms (72). Of note is the fact that diseases of the male gonad responsible for hypogonadism are not frequently present in male infertility. For instance, Klinefelter’s syndrome, the most frequent genetic cause of non-obstructive azoospermia (NOA), is found in a low percentage of infertile patients. In fact, NOA causes infertility only in 10-15% of infertile patients (73). For this reason, it is necessary to increase the research in the field of genetics and epigenetics (gene-environment interactions) of male infertility to provide the basis for the development of future etiology-based prevention and treatment (72). Undoubtedly not all causes of infertility are due to an endocrine disorder. However, the multidisciplinary approach with an endocrinologist expert in reproduction playing a central role is required for both the diagnosis and management of infertility.

To understand the economic impact of infertility on the healthcare system and patients, we have to consider the costs of a single ART cycle. The costs vary from $2,500 up to $10,000 based on the direct and indirect costs and also on the costliness of the underlying healthcare system. Direct costs include medical consultations, ovulation stimulation drugs, laboratory and embryology services, ultrasound scanning, oocyte retrieval, and embryo transfer, hospital charges, nursing and counseling services, and administrative and overhead charges. Indirect costs, on the other hand, are mainly related to the increased risk of multiple pregnancies associated with these procedures, resulting in an increment of the costs for the management of pregnancy and associated complications (74).

### Gender Identity Disorders

Gender identity disorders or gender dysphoria affects between 0.005 and 0.014% of biological males and 0.002-0.003% of biological females according to the DSM5 (75). Although infrequently, these disorders have a significant social and economic impact. Indeed, people with gender dysphoria require multidisciplinary teams to manage the process that will lead to gender reassignment. The main medical figures involved are endocrinologists for the management of hormone therapy, psychiatrist, considering the higher rate of depression that afflicts these patients, and the surgeon for permanent sex change. Moreover, the costs associated with possible complications of the treatment must also be considered. Indeed, both hormonal and surgical treatments are associated with greater risks of venous thromboembolism, bone mineral density, pubertal suppression, etc. (76).

## Pubertal Disorders

Disorders of puberty include premature puberty and pubertal delay. Precocious puberty is a condition characterized by the appearance of secondary sexual characteristics before the age of 8 years in girls and before 9 years of age in boys. It affects about 1/5000 children with a female-to-male ratio of 10:1 (77). Delayed puberty is a disorder that results in important psychological stress for both affected patients and their parents. It is characterized in girls by a lack of breast development after 13 years of age or in presence of a difference of more than 4 years between thelarche and menarche. In boys, it is characterized by a lack of testicular enlargement after the age of 14 or by a difference of more than 4 years between the onset of testicular development and the completion of the pubertal process. It affects approximately 2% of adolescents (78). About 60% of them has a constitutional delay in growth and puberty, 10% it is caused by central (hypothalamic or pituitary disease) hypogonadism, 7% relates to primary hypogonadism, and finally, 20% is due to functional forms of hypogonadism (e.g., forms associated with chronic systemic diseases) (49).

## Metabolic Diseases

The most prevalent metabolic alteration is represented by dyslipidemia and in particular by hypercholesterolemia. This condition is associated with a twice greater risk of developing cardiovascular events compared to unaffected patients (79). For this reason, adequate management is essential. In the United States, about 53% of adult men have elevated LDL levels (79). In Italy, according to data from the Heart Project of the Istituto Superiore di Sanità, 21% of men and 23% of women are hypercholesterolemic considering a population aged 34-74 years. In detail, 36.6% of men and 36.3% of women with hypercholesterolemia are unaware of their condition, 40.9% of men and 42.8% of women know they are hypercholesterolemic but are not treated, and 6.1% of men and 7.3% of women are aware of their condition but are not adequately treated (80). These data show that much remains to be done in the early diagnosis and management of these patients to prevent future cardiovascular events. Among the genetic forms, those with the highest prevalence are familial hypercholesterolemia (heterozygous variant), 1 case per 200-250 persons, and familial combined hyperlipidemia, 1 case per 100-200 (81).

The other metabolic condition with health, social, and economic implications is DM. This disorder affects more than 425 million people worldwide (prevalence of 8.5%), of which only 5-10% can be attributed to type I DM (82). Approximately 5.2 million deaths are attributable to DM globally, with a mortality rate of 82.4 patients per 100,000. In particular, cardiovascular mortality is responsible for 44% of deaths in patients with type 1 DM and 52% of deaths in type 2 DM (83). According to data from the 2019 ARNO Diabetes Observatory Report, between 6.2-7.2% of Italians are diabetic with about 4 million people with DM. However, another 1.5% appears to be undiagnosed, with approximately 1 million Italians unaware of being affected or not treated. Sixty-seven percent of DM cases involve patients over 65 years of age, 1% have less than 20 years of age, and 32% are of working age (20-64 years). This highlights the significant socio-economic implications of this disease (84). In detail, about 1 in 6 diabetics are hospitalized at least once a year. The hospitalization rate in diabetics is more than double that of non-diabetics with an average hospital stay of about 1.5 days longer in diabetics. The total cost of monitoring and treating DM is approximately $2,800, more than double that of a non-diabetic. Furthermore, the cost attributable to complications and comorbidities represents 90% of the total costs of the disease, while the management of the metabolic aspects only 10%. This highlights the importance of early diagnosis and treatment of the disease to prevent the onset of complications (84).

The main condition, which is partly associated with the metabolic alterations described above, is represented by obesity. The prevalence and incidence of this phenomenon are increasing due to evolutionary, biological, psychological, sociological, economic, and institutional factors (85). According to the WHO, more than 1.9 billion people were obese in 2016 (86). In Italy, about 25 million people are overweight and obese with a prevalence of obesity of 10.8% of the population (85). In 2017, overweight and obesity were responsible for 4.72 million deaths and 148 million years lived with disability reaching fourth place among the causes of death preceded only by hypertension, smoking, and hyperglycemia (85). Obesity is now responsible for a total cost of approximately 2 trillion dollars, which corresponds to 2.8% of the world gross domestic product with an impact on the global economy that overlaps that of smoking cigarettes. Excess body weight generates both significant direct costs, largely attributable to treatment and hospitalization for associated comorbidities, and indirect costs, related to loss of productivity due to illness and premature mortality (85).

## Neuroendocrine Tumors

Neuroendocrine tumors (NETs) are relatively rare tumors whose incidence, probably due to an improvement in diagnostic ability, seems to be increasing. In particular, the annual incidence varies from 3.4 cases per 100,000 inhabitants in Germany to 10.3 cases per 100,000 inhabitants according to the Kentucky registry (87). In Italy, from 1976 to 2010, the incidence of these tumors has increased from 0.7 to 5.3 cases per 100,000 inhabitants (88). To date, there is no simple and universally accepted classification criterion for NETs, although, according to the WHO, 3 different grades can be distinguished based on mitotic count or Ki67. However, according to the National Comprehensive Cancer Network (NCCN), sometimes the histological grade does not really correlate with their clinical and aggressiveness. Therefore, clinical evaluation plays a fundamental role in the most appropriate therapeutic management (89). Sometimes, NETs secrete hormones with resulting clinical manifestations. Among these, the carcinoid syndrome, due to serotonin secretion, is the most frequent one (90).

In most cases, NETs are sporadic, although in 20% of cases genetic abnormalities are associated with their occurrence. These include MEN 1 and 2, Von Hippel-Lindau (VHL) syndrome, neurofibromatosis, and tuberous sclerosis (89). NETs localize most frequently in the gastrointestinal tract (62-67%) and the lungs (22-27%) (89). In particular, NETs of the gastroenteropancreatic tract include pancreatic NETs that, in turn, may be nonfunctioning (approximately 60-90% of cases) or functioning. Among the latter, insulinomas (1-32 new cases per 1,000,000/year) and gastrinomas (0.5-21.5 new cases per 1,000,000/year) are the most frequent. Gastrinomas localize in the duodenal level in 70% of cases and 25% of cases at the pancreatic level. In addition, gastrinomas are associated with MEN1 in 20-25% of cases. Other functioning pancreatic NETs are extremely rare and may secrete vasoactive intestinal peptide (VIP), glucagon, somatostatin, or serotonin (carcinoid syndrome). Even rarer are those that secrete other hormones such as ACTH, GH, and parathyroid hormone (91).

Pulmonary NETs include typical and atypical carcinoids, large-cell neuroendocrine carcinomas (LCNEC), and small-cell lung carcinomas (SCLC). The latter is the most aggressive and also the most frequent among lung NETs with a prognosis of few months. It is also the one most frequently associated with paraneoplastic syndromes, such as syndrome of inappropriate antidiuretic hormone, Cushing’s syndrome, hypercalcemia from parathyroid hormone-related peptide secretion, and neurological syndromes (autoimmune neuropathies and encephalomyelitis) (89).

## Osteoporosis

Osteoporosis is associated with high morbidity and mortality. Each year, osteoporosis causes more than 8.9 million fractures, equivalent to one fracture every 3 seconds. In detail, 1 out of 3 women older than 50 years and 1 out of 5 men older than 50 years develop fractures from osteoporosis (92). In Europe, disability due to osteoporosis is higher than that caused by tumors (excluding lung cancer) and is comparable to or higher than that caused by chronic diseases, such as rheumatoid arthritis, asthma, and hypertension-related heart disease (92). In fact, fragility fractures rank fourth among causes of chronic morbidity after ischemic heart disease, dementia, and lung cancer but before chronic obstructive pulmonary disease and ischemic stroke. Moreover, hip fracture is associated with a 20% risk of mortality in the first 12 months after the event, whereas vertebral fractures are associated with an approximately 8-fold increase in age-adjusted mortality (93). In Italy, 3.2 million women and 0.8 million men have osteoporosis with a prevalence of 23.1% women and 7% men older than 50 years (93). The health care costs associated with fragility fractures are substantial. In Europe, considering Spain, Italy, France, Germany, United Kingdom, and Sweden, the cost amounts to approximately 37 billion dollars. In the United States and China, the costs are approximately $22 billion. In Italy, in 2017, health expenditure was approximately $9.4 billion (94).

## Multiple Endocrine Neoplasia Syndromes

MEN syndromes are characterized by the occurrence of multiple neoplasms of endocrine glands that can be benign and malignant. There are basically 3 main variants (MEN 1, MEN 2A, and MEN 2B) and some new entities, such as MEN 4 considered a variant of MEN 1 and familial medullary thyroid cancer (FMTC) considered a variant of MEN 2A (95).

MEN 1 is rare, with an estimated prevalence of 2-3 cases per 100,000 people. It is due to mutations of the *MEN1* gene that encodes for menin, a protein involved in the regulation of cell proliferation and differentiation, apoptosis, endocrine-metabolic functions, and maintenance of genomic stability through DNA repair. The endocrine glands more frequently involved in the neoplastic transformation are parathyroids, pancreatic islets, and anterior pituitary gland. In particular, the main disease present in almost 100% of the patients is primary hyperparathyroidism. Gastroenteropancreatic endocrine (GEP) tumors occur in 70-80%. In more than half of them, they are gastrinomas that, along with foregut carcinoids, are the leading cause of morbidity and mortality in these patients. Finally, pituitary tumors are found in 10-60% of patients and mainly they are prolactin-secreting pituitary macroadenomas (95–96).

MEN 2 is rarer than MEN 1. In fact, the estimated prevalence of all variants combined is about 1 case per 30,000 people. It is associated with mutations of the RET proto-oncogene encoding for a transmembrane receptor tyrosine kinase that physiologically, upon ligand binding, dimerizes initiating downstream pathways. When mutated, the receptor is constitutively active. The main manifestation of all three variants is medullary thyroid carcinoma, which unlike sporadic forms is usually bilateral, multicentric, and associated with C-cell hyperplasia (preneoplastic lesion). In addition to MTC, patients with MEN 2A can develop pheochromocytoma (also bilateral) in 50% of the cases and parathyroid hyperplasia in 25% of the cases. More rarely, amyloid cutaneous lichen (10%), generally in the interscapular region, and Hirschsprung’s disease (2%) (agangliosis of the intestine) may be present. On the other hand, in patients with MEN 2B, in addition to MTC, pheochromocytoma can be observed in about 50% of cases, and in almost 100% of cases, there is marfanoid habitus and mucosal neurinomas. In FMTC, MTC is present alone (95, 96).

The analysis of the RET gene is crucial not only for the diagnosis of MEN 2 but also for the prognostic role given the important genotype-phenotype association present in this disease. In fact, MEN 2A is the most frequent form and is associated with an intermediate aggressiveness of the disease. MEN 2B is the rarest but also the most aggressive. Finally, FMTC is considered the mildest variant since it does not present the other manifestations of MEN 2A (95–97).

The QoL of patients with these syndromes is obviously impaired. Particularly in patients with MEN 1, the persistence of hyperparathyroidism after surgery, the need for numerous medical appointments, and sometimes the need to travel to reach centers specialized in the treatment of these rare diseases, represent the factors that mostly affect the QoL of these patients (98). In addition, the economic burden that these patients often have to bear together with the need to leave work for medical care contribute to aggravate the distress (99). In MEN 2, on the other hand, symptoms related to the presence of pheochromocytoma and/or parathyroid hyperplasia, possible complications associated with thyroidectomy and lymphadenectomy (hypothyroidism, laryngeal nerve palsy, and spinal accessory nerve dysfunction), and the use of tyrosine kinase inhibitors in patients with metastatic disease have been associated with significant QoL worsening (100).

## Autoimmune Polyendocrine Syndromes

APS is characterized by the development of multiple autoimmune endocrine diseases associated or not with autoimmune disease of non-endocrine organs. The association between these diseases is not random but configures 4 main clinical pictures. In detail, APS-1 is mainly characterized by the presence of at least two among chronic candidiasis, chronic hypoparathyroidism, and Addison’s disease. APS-2 associates Addison’s disease with autoimmune thyroid diseases, and/or type 1 DM. APS-3 is characterized by autoimmune thyroid diseases associated with other autoimmune diseases (excluding Addison’s disease and/or hypoparathyroidism). Finally, APS-4 presents combinations not included in the previous groups (101).

APS-1 is a monogenic disease due to a mutation in a gene called autoimmune regulator with a prevalence ranging from 1:9000 inhabitants among the Iranian Jewish community, 1:25,000 in Finland, 1:80,000-90,000 in Norway (101). In Italy, there is wide regional variability in the prevalence of this disease that about 1:14,400 in Sardinia and 1:200,000 in northern Italy (102).

APS-2 is more common than APS-1 with an estimated incidence of 1.4-4.5 cases per 100,000 inhabitants. It predominantly affects women (F/M ratio 2.7-3.7) and is very rare in children (101). Since autoimmune thyroid diseases are quite frequent, affecting 7-8% of the general population, and considering that in the study by Betterle and colleagues in about half of these patients there is another autoimmune disease that does not include Addison’s disease and chronic hypoparathyroidism, we can estimate a prevalence of APS-3 to be 3.5-4% of the total population (101).

APS-4 is rare and there are no studies that have evaluated its true incidence and prevalence (101).

Another condition is the X-linked immune dysregulation, polyendocrinopathy, and enteropathy (IPEX), an extremely rare inherited syndrome characterized by early-onset type 1 DM, intractable diarrhea, and malabsorption due to autoimmune enteropathy and various forms of dermatitis (eczematiform, ichthyosiform, or psoriasiform). It is due to mutation of the FOXP3 gene expressed in regulatory T cells involved in the control of the immune response. It occurs in childhood with a prevalence of about 1,000,000 inhabitants (103).

APS are diseases with considerable clinical impact since they require multidisciplinary interventions, multiple hormone replacement therapies, and the management of complications such as adrenal (APS-1 and 2) and hypocalcemic crises (APS-1), cancer of the esophagus and mouth (APS-1), complications of DM (APS-2), and infections (IPEX). Certainly, there is a need for further research in this area, especially to identify genetic mechanisms and environmental triggers to tailor the therapy to the patient and identify immunomodulatory treatments that block the autoimmune process before the irreversible organ damage occurs (103).

## Endocrine Hypertension

Hypertension is a disorder that affects approximately 30% of the adult population. In most cases, hypertension is primary, also referred to as essential, whereas it is secondary in about 10% of cases. More than 15 endocrine diseases can cause secondary hypertension. These include primary aldosteronism, pheochromocytoma, Cushing’s syndrome, thyroid disease, and hyperparathyroidism, acromegaly, and mineralocorticoid excess. Primary hyperaldosteronism is the most frequent form believed to be responsible for 5% of all cases of hypertension and 20% of cases of hypertension resistant to drug therapy (104). For the epidemiological data of each of these diseases, please refer to the respective paragraphs.

## Endocrine Diseases and COVID-19

Knowledge suggests on the correlation between endocrine disorders and COVID-19 is still quite scarce. Some evidence suggests a strong correlation between SARS-CoV-2 infection and DM. Indeed, SARS-CoV-2 infection aggravates inflammation and alters immune system responses, leading to difficulties in glycemic control and worsening insulin resistance. The viral infection increases also the risk of thromboembolism and cardiorespiratory failure more in patients with DM than in patients without it. This helps, in part, to explain the poor prognosis of patients with DM (105).

The sex-related difference in the severity of COVID-19 has an endocrine explanation. Indeed, host entry of SARS-CoV-2 is mediated by transmembrane serine protease 2 (TMPRSS2), whose transcription is, in turn, promoted by the activation of the androgen receptor. For this reason, men have a more facilitated viral entry into host cells. Accordingly, conditions associated with increased sensitivity of the androgen receptor, such as androgenetic alopecia and prostate cancer, have been correlated with worse COVID-19 outcomes and hospitalization (106). Furthermore, in patients with prostate cancer, androgen deprivation therapy seems to have a protective role in contracting the infection and mitigating the COVID19 course (107). This evidence might explain both the low fatalities observed in prepubertal children and the differences between sexes regarding SARS-CoV-2 infection (106). Therefore, it could be hypothesized that in hypogonadal patients with infection, testosterone replacement therapy could be administered at a lower dosage than generally used. However, it must also be taken into account that the viral infection itself may alter Leydig function thus compromising testosterone secretion. Moreover, the systemic inflammatory response is greater in patients with hypogonadism due to a lack of the immunosuppressive effects of testosterone. This could modify the duration and course of the infection in these patients (108).

The correlation between sexual function, particularly ED and COVID-19 has also been recently investigated. In fact, Sansone and colleagues in a study conducted on 100 patients, showed that 25 patients who were infected with SARS-CoV2 had a higher prevalence of ED than the 75 healthy patients who were not infected. Furthermore, regression analysis confirmed that COVID-19 has a significant effect on the development of ED independently of other variables affecting erectile function, such as psychological status, age, and BMI (109). This association is probably due to the detrimental effect of the disease on endothelial function, whose integrity is, in turn, fundamental for the erective mechanism. In addition, the impairment of pulmonary function, as well as the significant psychological impact that the SARS-CoV 2 pandemic has had on the population, are factors that may contribute to the genesis of ED (110). On the other hand, the evidence on the presence of the virus in the seminal fluid is still conflicting (111). A recent study has shown an increased risk for patients with COVID-19 to develop oligo-crypto-azoospermia after recovery from the disease, suggesting the usefulness of requesting sperm analysis to patients of reproductive age who were affected by COVID-19 (112).

Evidence on COVID-19 and pituitary and adrenal function are still scarce. Undoubtedly, patients with AI require more careful monitoring and increased doses of the replacement therapy in case of SARS-CoV-2 infection. However, to date, there is no evidence of direct pituitary or hypothalamic effects by COVID-19 (113). The possibility that this association exists comes from previous evidence on SARS. In fact, 40% of patients with SARS were reported to have central AI probably due to pituitary dysfunction. The insufficiency, however, resolved within one year after the infection (114). Since there is an important homology between SARS and COVID-19 viruses, it can be hypothesized a similar association between SARS-CoV-2 infection and the risk of developing AI (113, 115).

Finally, a recent systematic review of 9 reviews concluded that patients with COVID-19 may develop thyroid dysfunction that includes thyrotoxicosis, hypothyroidism, and non-thyroidal illness syndrome. However, it is not clear whether this association is directly attributable to the virus or is merely a consequence of the disease and/or its treatment. In fact, the use of heparin can increase the levels of non-esterified fatty acids that displace thyroxine and triiodothyronine from the binding proteins, leading to increased levels of FT4 and FT3. Corticosteroid therapy can also decrease TSH levels, reduce thyroxine-binding protein levels resulting in increased FT4 levels, and inhibit the conversion of FT4 into FT3. Furthermore, patients with thyroid disease do not have an increased risk of SARS-CoV-2 infection and do not require COVID-19-adapted follow-up. Some cases of subacute thyroiditis have been reported with a late consequence after SARS-CoV-2 infection, but further studies are needed to better clarify this aspect (116).

## Endocrine Diseases During Pregnancy

**Figure 1:**
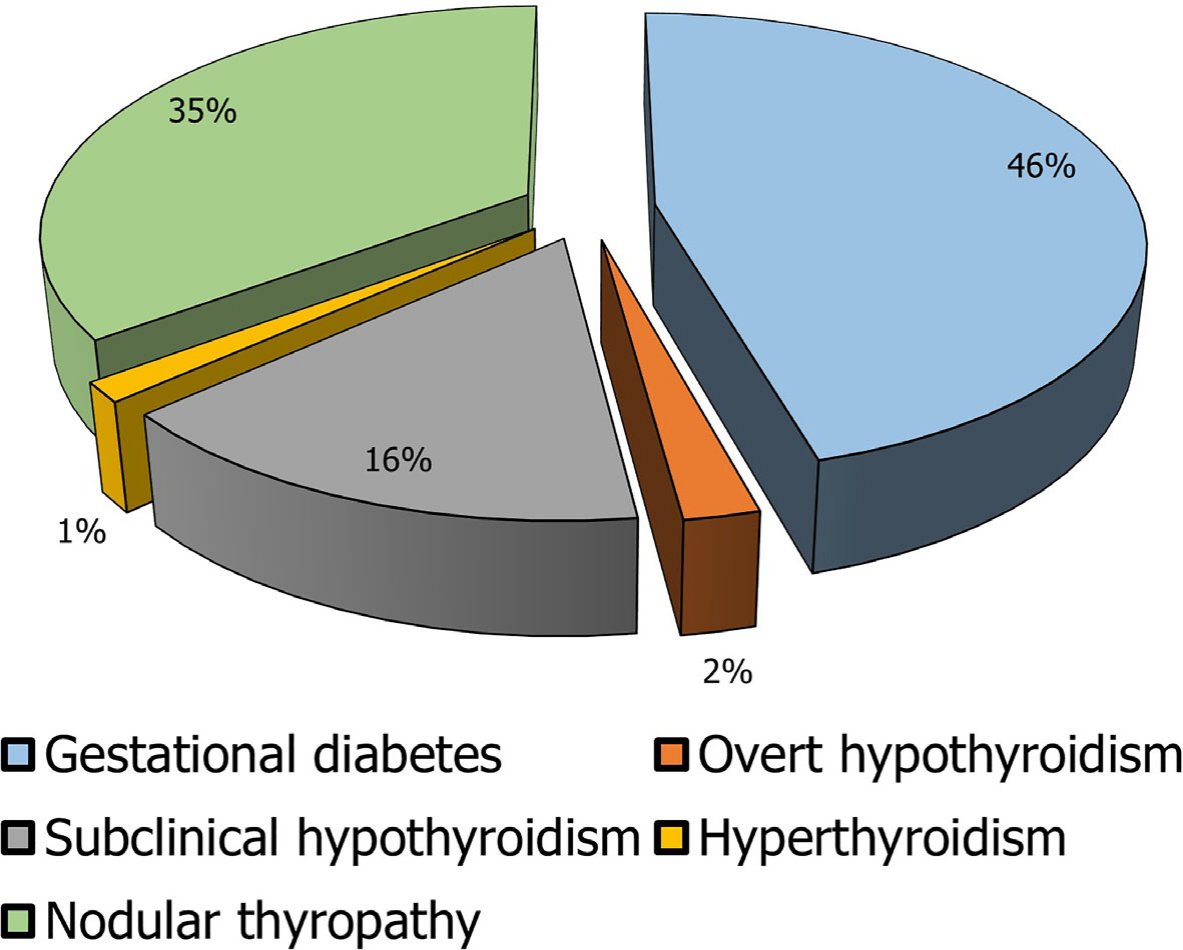
Prevalence of the main endocrine diseases in pregnancy. In the elaboration of the pie chart, the prevalence data have been applied to the number of pregnancies in 2018 in Italy calculated as the sum of the number of deliveries, the number of miscarriages, and the number of voluntary interruptions of pregnancy (117, 118).

The most frequent endocrine diseases in pregnancy are gestational DM and thyroid diseases **(** **Figure 1** **)**. In detail, gestational DM affects about 7% of pregnancies and its inadequate management is associated with important maternal-fetal complications such as gestational hypertension, placental abruption, intrauterine growth retardation with intrauterine death, and congenital malformation (119).

In line with the general population, the thyroid disease with the highest prevalence is thyroid nodule. Indeed, its diagnosis is made in about 5% of pregnancies. However, given the low percentage of thyroid nodules that turn out to be malignant, their finding during gestation seldom requires urgent surgery or termination of pregnancy (120). In contrast, thyroid dysfunctions are not common but clinically more severe. Gestational hyperthyroidism occurs in 1-2 pregnancies/1000 with a prevalence of about 0.2%. The main causes of gestational hyperthyroidism are similar to those of the general population except for the forms caused by an excess of human chorionic gonadotropin. In pregnant women, hyperthyroidism can promote gestational hypertension, miscarriage, and placental abruption, while in the newborn it can lead to low birth weight, prematurity, neonatal hyperthyroidism, and intrauterine death (120).

Hypothyroidism, on the other hand, has a higher prevalence. In its subclinical form, it affects 2.2% of pregnant women, while overt hypothyroidism has a frequency in 0.3% of pregnancies. Hypothyroidism also promotes gestational hypertension, placental abruption, low birth weight, postpartum hemorrhage, congenital malformations, and intrauterine death (120).

The prevalence of other endocrine disorders in pregnancy is extremely low since many endocrine diseases can alter the ovulatory process. For example, pituitary disorders are quite rare in pregnancy since both pituitary hyperfunction (prolactinomas, acromegaly, Cushing’s disease, etc.) and hypofunction could compromise fertility. Indeed, very rare are the cases described in the literature of acromegalic women that got pregnant since the excess of growth hormone causes anovularity. The first case of normal pregnancy in an acromegalic patient was described in 1954. Hypopituitarism is also extremely rare and the few cases described in the literature refer to women who have undergone ART cycles (121).

The prevalence of primary hyperparathyroidism in pregnancy is around 0.15%. Hypoparathyroidism is also rare occurring mainly as a consequence of thyroidectomy. As evidence of the rarity of the event, the first case of hypoparathyroidism in pregnancy was described in 1942 (121).

As far as adrenal diseases, to date, about 200 cases of Cushing’s syndrome have been described in pregnant women (both ACTH-dependent and ACTH-independent). The rarity of this disease in the gestational period derives from the fact that the hypercortisolism and hyperandrogenism present in Cushing’s syndrome alter fertility. Addison’s syndrome is also quite rare with a prevalence of approximately 1 case per 30,000 pregnancies. Even rarer is pheochromocytoma with a prevalence of 0.007% of pregnant women (121).

## Conclusions

**Figure 2:**
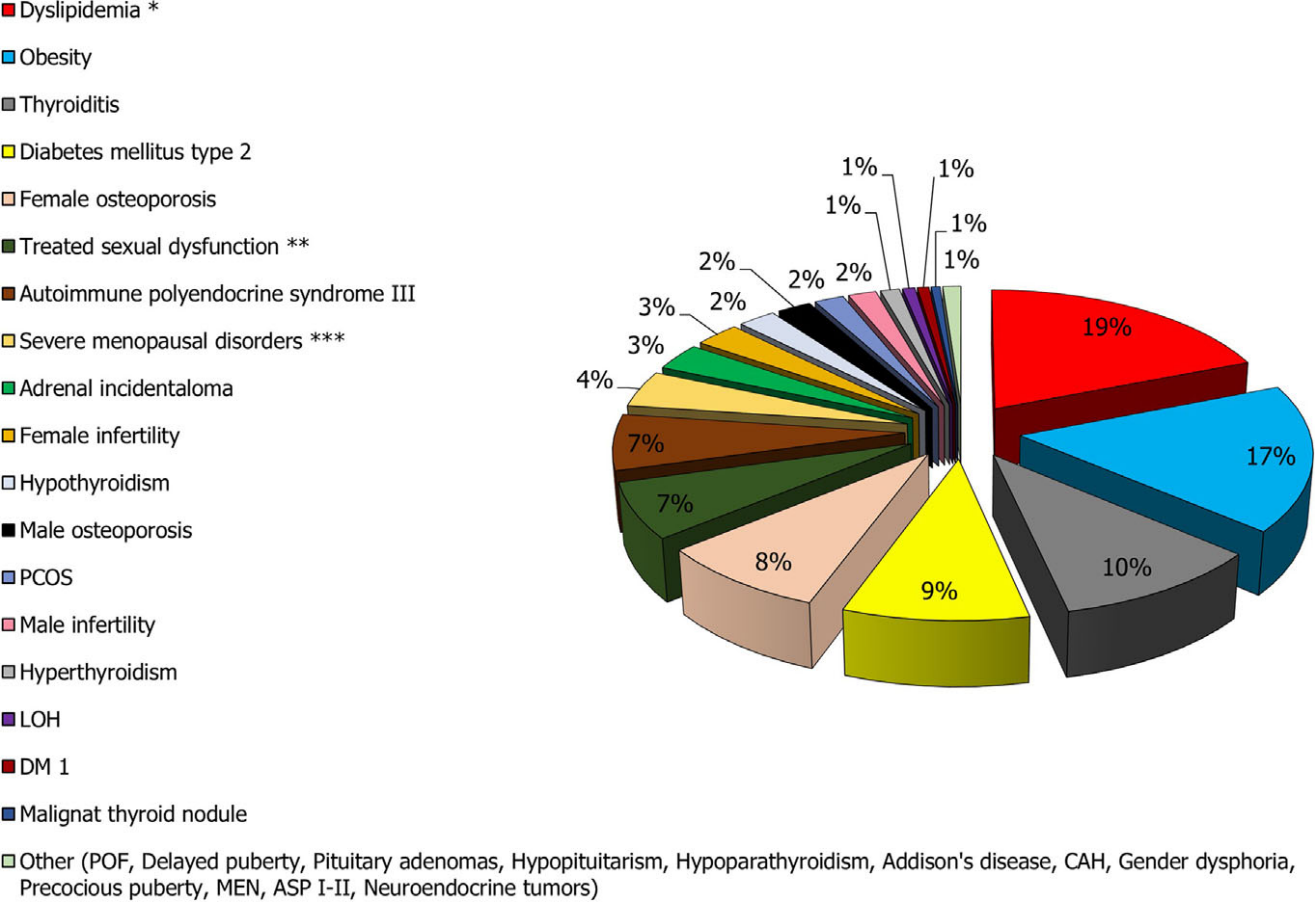
Prevalence of the main endocrine diseases in the Italian population. *The prevalence of hypercholesterolemia (the most frequent condition) was considered in the calculation. **The total prevalence of sexual dysfunction is much higher than that of other conditions such as dyslipidemia and obesity. However, only 7% of men and women with sexual dysfunction are treated or otherwise see a physician for treatment of the disorder. ***Menopause affects approximately 10,000,000 women. Of these, 80% report complaints related to the condition, but only 25% seek medical attention.

Endocrine dysfunctions are chronic diseases with a relevant negative impact on the patients affected and considerable social and economic burden for the health system. Taken into account the data of prevalence above-reported to the Italian population or, for some diseases, to specific reference populations (boys <9 years and girls <8 years for precocious puberty, adolescents with delayed puberty, postmenopausal women for menopausal disorders, women of childbearing age for female infertility and polycystic ovary syndrome, women aged 15-40 years for premature ovarian failure, and women and men >50 years for osteoporosis), we evaluated the real epidemiological impact of the various diseases of endocrine interest **(** **Table 2** and **Figure 2** **)**. In particular, based on the graphical elaboration of the data, it does not a surprise that metabolic diseases have the greatest impact on clinical practice and economic costs. Therefore, the role of the endocrinologist in the adequate management of these patients is prominent.

**Table 2:** List of major endocrine diseases (in decreasing order of frequency).

	Disease
**1**	Ultrasound detectable thyroid nodule
**2**	Obesity
**3**	Dyslipidemia
**4**	Thyroiditis
**5**	Diabetes mellitus type 2
**6**	Female osteoporosis
**7**	Treated sexual dysfunction
**8**	APS III
**9**	Menopausal disorders
**10**	Female infertility
**11**	Hypothyroidism
**12**	Male osteoporosis
**13**	PCOS
**14**	Male infertility
**15**	Hyperthyroidism
**16**	Adult-onset hypogonadism (LOH)
**17**	Diabetes mellitus type 1
**18**	Malignant thyroid nodule
**19**	Hyperparathyroidism
**20**	POF
**21**	Delayed puberty
**22**	Pituitary adenoma
**23**	Hypopituitarism
**24**	Hypoparathyroidism
**25**	Addison’s Disease
**26**	APS II
**27**	Congenital Adrenal Hyperplasia
**28**	Gender Dysphoria
**29**	MEN
**30**	Early Puberty
**31**	APS I
**32**	Neuroendocrine tumors
**33**	IPEX

**Figure 3:**
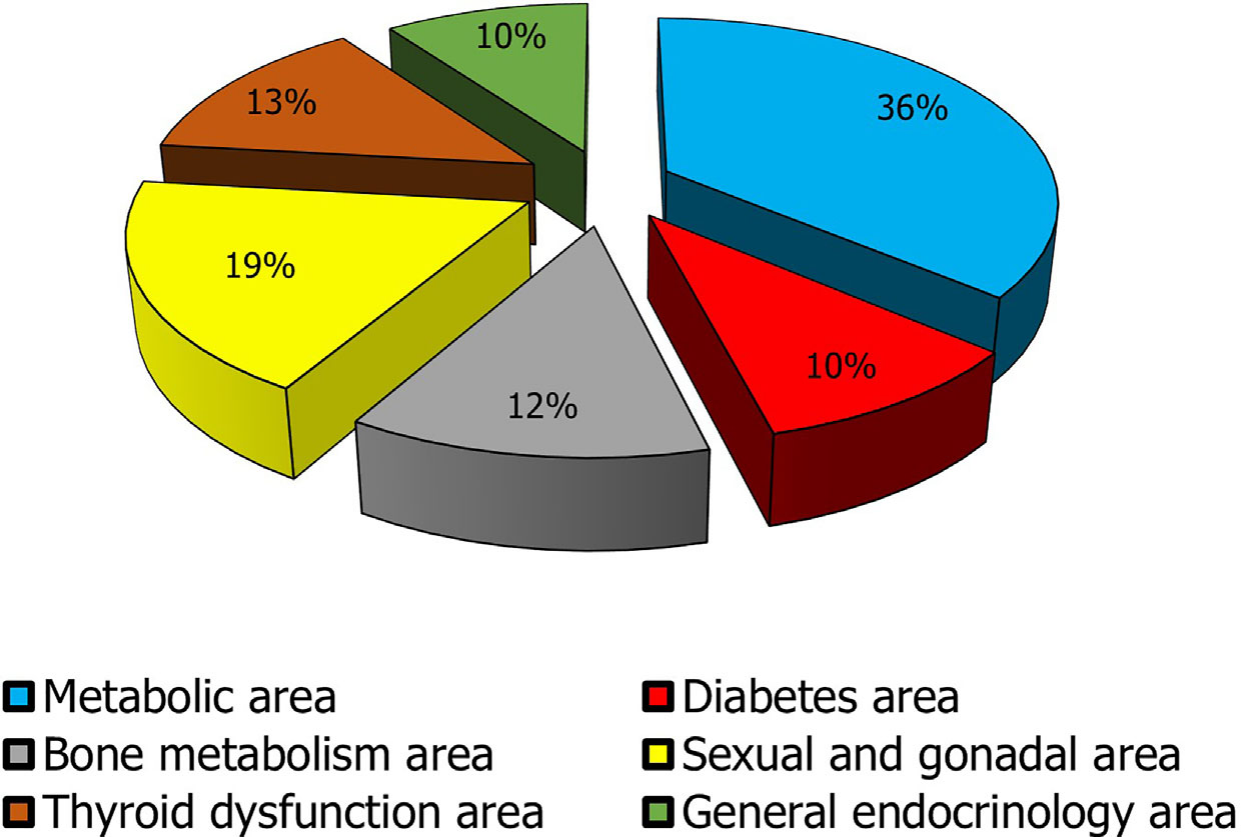
Impact of the macro-areas of endocrine interest in clinical practice. The metabolic macro-area is given by the sum of obesity and dyslipidemia data; the diabetes mellitus area encloses DM1 and DM2 data; the bone metabolism area collects prevalence data of male and female osteoporosis, hypoparathyroidism, and hyperparathyroidism; The sexual and gonadal area includes data on male and female infertility, LOH, PCOS, POF, severe menopausal disorders, treated male and female sexual dysfunction, early and delayed puberty, and gender dysphoria; Thyroid dysfunction area includes thyroiditis, hypothyroidism, hyperthyroidism, and malignant thyroid nodule (the prevalence of ultrasonographic nodular pathology was not considered to avoid to overestimate the problem. In fact, the most clinically significant nodules are those that are malignant on cytologic and/or histologic examination); General endocrinology area encompasses data regarding pituitary adenomas, hypopituitarism, adrenal incidentaloma, Addison’s disease, CAH, neuroendocrine tumors, ASP, MEN.

Gonadal and sexual disorders rank second in this list **(** **Figure 3** **)**. As above-mentioned considering the close associations of these conditions with systemic diseases and that sometimes they may escape diagnosis and treatment, the importance of andrological and gynecologic endocrinology results clear in recognizing these patients to improve their QoL and to prevent future morbidity.

## Author Contributions

Conceptualization: AC and SV. Writing—original draft preparation, AC. Writing—review and editing, AEC, AA, SV. Visualization: EG. Data curation: LM, RC, EG. Supervision, RAC, AA. Project administration, AEC and SV. All authors contributed to the article and approved the submitted version.

## Conflict of Interest

The authors declare that the research was conducted in the absence of any commercial or financial relationships that could be construed as a potential conflict of interest.
